# Moral distress among pediatric nurses: a cross-sectional study from Sichuan, China

**DOI:** 10.3389/fped.2026.1787502

**Published:** 2026-04-29

**Authors:** Li Li, Yulan Shi, Menglin Tang

**Affiliations:** 1 Outpatient Department, West China Hospital, Sichuan University, Chengdu, China; 2 Department of Critical Care Medicine, West China Hospital, Sichuan University, Chengdu, China; 3 West China School of Nursing, Sichuan University, Chengdu, China; 4 Department of Cardiovascular Surgery, West China Hospital, Sichuan University, Chengdu, China

**Keywords:** influencing factors, moral distress, nurses, nursing ethics, pediatrics

## Abstract

**Background:**

Moral distress is common among pediatric nurses. However, evidence on moral distress in this population from non-Western healthcare settings remains limited.

**Objectives:**

This study aimed to assess the level of moral distress among pediatric nurses in China and identify factors influencing moral distress in this population.

**Methods:**

A cross-sectional survey was conducted from June to August 2024. Using census sampling, all 29 tertiary A hospitals in Sichuan Province, China, concurrently operating the Pediatric Intensive Care Units (PICUs), Neonatal Intensive Care Units (NICUs), and general pediatric wards were selected. Questionnaire invitations were sent to registered pediatric nurses at these hospitals, and 1,292 nurses meeting the inclusion criteria participated. The valid response rate was 76.8%. Data were collected using a demographic questionnaire, the Revised Moral Distress Scale–Nurse Pediatric Version (MDNPV), the Hospital Ethical Climate Survey (HECS), and the Moral Sensitivity Questionnaire-Revised (MSQ-R). Data analysis employed the Wilcoxon Rank-Sum Test, Kruskal–Wallis H test, Spearman rank correlation, and multiple linear regression.

**Findings:**

The median total moral distress score was 64.00 (IQR: 36.00–84.00). Compared with nurses working in general pediatric units, PICUs and NICUs reported higher levels of moral distress (*H* = 30.828, *P*<0.01). Those with the title of charge nurse and above experienced higher levels of moral distress than nurses with lower titles (*H* = 17.620, *P*<0.01). Female nurses experienced more severe moral distress than male nurses(*Z* = 2.006, *P*<0.05). Multiple linear regression identified six independent predictors of moral distress: department, professional title, gender, hospital ethical climate, moral sensitivity, and nurse–physician collaboration, collectively accounting for 40.2% of the variance (*R*^2^ = 0.402, adjusted *R^2^* = 0.398, *P* < 0.05).

**Conclusion:**

In our study, moral distress was prevalent among pediatric nurses in Sichuan Province, China. The levels of moral distress were associated with department, professional title, gender, hospital ethical climate, moral sensitivity, and nurse–physician collaboration.

## Introduction

Moral distress was first introduced into nursing by Jameton (1984), who defined it as occurring “when one knows the right thing to do, but institutional constraints make it nearly impossible to pursue the right course of action.” (1) This phenomenon is prevalent across healthcare disciplines, with nurses experiencing the highest frequency and severity of moral distress (2, 3), particularly those working in intensive care and pediatric settings (4, 5). Repeated exposure to morally distressing situations alters the ease stage of moral reckoning among nurses, causing psychological and behavioral disturbances, and thereby requiring them to take a stand or give up (6). Orgambídez et al. (7) demonstrated that moral distress is strongly correlated to emotional exhaustion in healthcare professionals. Hancock et al. (8) found that the frequency of moral distress was negatively correlated with the level of nursing skills and the quality of nursing services. A study showed that moral distress was negatively correlated with nurses’ job satisfaction and work engagement and positively correlated with burnout and the intention to leave (9, 10). In response, the American Association of Critical-Care Nurses has identified moral distress as a “frequently overlooked problem” and introduced the standardized “4A” process (Ask, Affirm, Assess, and Act) for its management (11). Despite this recognition, it remains a persistent challenge in nursing practice worldwide, and continues to be a focus of growing academic investigation (12).

In pediatric settings, end-of-life and complex ethical issues are frequent. Children's physical and mental immaturity often limits their capacity to participate in healthcare decisions, making them dependent on legal guardians. The recurring challenge of aligning nursing interventions with the child's best interests leads to frequent and demanding ethical decisions (13). Available evidence underscores the global prevalence of this issue. In Canadian NICUs and PICUs, 8.3% of healthcare providers reported significant work-related moral distress (14). Australian studies revealed that 72% of NICU nurses experienced morally distressing events monthly (15), while 58% of NICU/PICU staff encountered moral distress, with nurses showing the highest levels (16). Similarly, 6% of staff at Nordic pediatric cancer centers considered leaving due to moral distress (17). These findings collectively highlight the critical need to address moral distress among pediatric nurses, particularly those in PICUs and NICUs.

Research on moral distress is increasing annually, with approximately 71% of studies focusing on nurses (3). However, a systematic review by Prompahakul & Epstein (18) of literature published between 1999 and 2019 revealed that of the identified studies on moral distress in nurses, 156 were conducted in Western regions such as North America, Canada, Europe, and Australia, while only 16 originated from non-Western countries, including those in the Middle East and Asia. This indicates a notable lack of data on moral distress among nurses in non-Western contexts. Among the limited studies from these regions, one investigation involving 465 nurses in Shandong Province, China, confirmed that Chinese clinical nurses also experience moral distress (19). However, pediatric nurses were not examined separately in that investigation. Therefore, the present study proposes that pediatric nurses in China experience moral distress, with levels varying by unit type and being more severe in PICUs and NICUs. To test this premise, we conducted a large-scale, multicenter cross-sectional survey of pediatric nurses at all tertiary hospitals in Sichuan Province that concurrently operate PICUs, NICUs, and general pediatric wards. This study aimed to: (1) assess the level of moral distress among pediatric nurses in Sichuan, China; and (2) identify factors influencing moral distress in this population, thereby providing an evidence base for developing tailored intervention strategies.

## Methods

### Design and sample

This observational cross-sectional study enrolled pediatric nurses from 29 Level A tertiary hospitals in Sichuan Province, located in western China, between June and August 2024. In China, hospitals are classified into three categories: primary, secondary, and tertiary. Each level is further subdivided into three grades—A, B, and C—based on the quality of medical care, medical education, and medical research provided. It is noteworthy that three level-A tertiary hospitals represent the highest tier in China and feature the most comprehensive range of departments. The aim was to investigate the level of moral distress among pediatric nurses in Sichuan Province, and we hope to compare differences across three pediatric departments: PICUs, NICUs, and general pediatric wards. To achieve this, we consulted data from the official website of the Sichuan Provincial Health Commission; there are 105 Class III Grade A hospitals in the province. We individually reviewed the official websites of each of the hospitals to verify whether it concurrently operated PICUs, NICUs, and general pediatric wards. Finally, 29 hospitals met the criteria. We next identified the number of registered nurses working in the relevant departments of these hospitals. For hospitals where this information was not publicly available online, the research project manager confirmed the nurse counts through direct consultation with the nursing departments during the formal data collection process. In total, 1,682 pediatric register nurses were identified as the target population.

The study applied inclusion and exclusion criteria for participant selection. The inclusion criteria were as follows: (1) full-time healthcare workers, (2) having at least one year of frontline clinical experience in pediatrics, and (3) providing informed consent and voluntarily agreeing to participate. The exclusion criteria included (1) nurses visiting from other hospitals for further education and (2) nurses temporarily absent from their positions during the study period due to illness or maternity leave.

An online survey platform was used to conduct the study (https://www.wjx.cn/), thereby minimizing privacy risks associated with paper-based questionnaires. The project manager contacted the nursing directors of the 29 participating hospitals via email to obtain institutional approval and requested permission to post recruitment notices and questionnaire links on the nursing departments’ online platforms. Following approval, text messages were sent to pediatric ward nurses to alert them to the recruitment notice. The online survey remained accessible for a 14-day period. Before completing the survey, each participant received 15–20 min of online training covering the study's purpose, content, procedure, and important considerations. The survey system was configured to prevent submission if any required information was omitted; participants were automatically redirected to incomplete sections, ensuring all items were filled out before final submission. In addition, backend settings restricted each IP address to a single response to prevent duplicate submissions. Two researchers monitored the incoming data in the backend, and data were exported after no new entries were recorded for one week. Two additional researchers reviewed the questionnaire responses for quality control, removing any that contained errors or contradictions. In addition, the authors adhered to the Strengthening the Reporting of Observational Studies in Epidemiology (STROBE) reporting checklist for observational studies.

### Sample size estimation

In regression analysis, the sample size should satisfy *N* ≥ 20 × m, where N represents the total sample size and m denotes the number of independent variables. This study included 33 independent variables, yielding a minimum sample size of 660. Accounting for an estimated 20% attrition rate, the target sample size was increased to at least 825 pediatric nurses.

### Ethical considerations

The study received approval from the Bioethics Committee of West China Hospital, Sichuan University (Approval No. 2021-1783) before commencement. All participants were informed about the study purpose, procedures, and methodology prior to participation. Questionnaires were administered only after participants provided fully informed consent. Participation was voluntary, and respondents could withdraw at any time without penalty. The survey was conducted anonymously, and all data were stored on an encrypted computer accessible exclusively to the research team.

### Measures

In the preliminary phase of the study, the research team completed a systematic review of factors potentially influencing nurses’ moral distress. The selection of independent variables in this study was informed by the findings of the prior research.

For details on the prior research findings, see Additional file 1: Appendix 1.

### Personal and professional characteristics

This section comprises two parts: demographic characteristics and subjective perceptions of nursing care. Demographic characteristics included department (general pediatric ward, PICU, NICU), age (continuous), gender (male, female), education level (associate degree or below, bachelor's degree, master's degree or above), marital status (unmarried, married, divorced), number of children (none, one, two, three or more), years in practice (<5, 5–10, >10 years), professional title (primary title, middle title, vice-senior title and above), monthly income (from <4,000 RMB to >10,000 RMB, in four categories), and average number of night shifts per month (from 0 to >10, in four categories). Subjective perceptions were assessed using yes/no items covering adequacy of nurse staffing, job satisfaction, autonomy in decision-making, participation in hospital management, and ethics training.

### Moral distress

The Revised Moral Distress Scale–Nurse Pediatric Version (MDNPV), developed by Hamric (20), was used in this study to assess moral distress among pediatric nurses. The Chinese version, translated and culturally adapted by Zhu in 2020 (21), consists of 21 items. Each item is rated on a 5-point Likert scale for frequency (0 = never to 4 = very frequently) and intensity (0 = none to 4 = great extent). The score for each item was calculated by multiplying the frequency and intensity ratings. The total MDNPV score, ranging from 0 to 336, was obtained by summing all item scores, with higher scores indicating greater moral distress. Two questions additionally elicited nurses’ intent to leave their clinical positions: “Have you ever left or considered leaving a clinical position due to moral distress?” and “Are you currently considering leaving your position due to moral distress?” The scale demonstrated good psychometric properties: the content validity index (S-CVI) was 0.90, split-half reliability was 0.87, and test-retest reliability was 0.91.

### Hospital ethical climate

The Hospital Ethical Climate Survey (HECS) was developed by Olson in 1998 (22). It is applicable to nurses at the level of ethical climate in hospitals. The Chinese version of the scale was translated by Wang in 2018 (23). This 26-item instrument employs a 5-point Likert scale, with total scores ranging from 26 to 130. The scale includes the five dimensions of relationships with peers, patients, managers, the hospital, and physicians. Higher scores reflect a more positive ethical climate as perceived by nurses. The Chinese version demonstrated good psychometric properties: Cronbach's *α* was 0.915, test-retest reliability was 0.786, I-CVI values ranged from 0.83 to 1.00, and S-CVI was 0.89.

### Moral sensitivity

The Moral Sensitivity Questionnaire-Revised (MSQ-R), developed by Lützén in 2006 (24), was used to assess moral sensitivity among pediatric nurses. The Chinese version of the scale (25) consists of nine items across two dimensions: moral responsibility and strength and sense of moral burden. Each item is rated on a 6-point Likert scale, yielding total scores from 9 to 54, with higher scores reflecting greater moral sensitivity. It demonstrated satisfactory internal consistency, with a Cronbach's *α* of 0.82 and item-total correlations ranging from 0.524 to 0.717. The questionnaire has been validated for use with Chinese nurses.

### Nurse-physician collaboration

The Nurse-Physician Collaboration Scale (NPCS), developed by Ushiro in 2009 (26), was used to evaluate collaboration levels among pediatric nurses. The Chinese version, adapted by Chen in 2014 (27), contains 21 items rated on a 5-point Likert scale, with total scores ranging from 21 to 105. The scale includes the three dimensions of joint participation in the cure/care decision-making process, sharing of patient information, and cooperativeness. Higher scores reflect more positive perceptions of collaboration. The scale's validity and reliability are well established, with an S-CVI/Ave of 0.913, Cronbach's *α* of 0.946, Guttman's split-half coefficient of 0.881, and test-retest reliability of 0.713, supporting its use with Chinese nurses.

### Preliminary survey

A pre-survey was conducted prior to the formal investigation to assess the reliability of the instruments. A convenience sample of 45 pediatric nurses who met the inclusion and exclusion criteria was recruited from the PICU of a tertiary Grade A general hospital in Chengdu, Sichuan Province. All scales demonstrated good reliability: Cronbach's *α* was 0.970 for the MDNPV, 0.969 for the HECS (with its five dimensions ranging from 0.759 to 0.918), 0.934 for the MSQ-R (with its two dimensions being 0.887 and 0.943), and 0.987 for the NPCS (with three dimension coefficients ranging from 0.940 to 0.983). These results supported the suitability of the questionnaire for use in the study.

### Statistical analyses

Statistical analyses were conducted using SPSS 25.0. Descriptive statistics, including frequencies, percentages, means, standard deviations, medians, and interquartile ranges, were computed to summarize the sample characteristics. Group differences in moral distress across demographic characteristics were examined using the Wilcoxon rank-sum test and the Kruskal–Wallis H test. Spearman's rank correlation was used to assess relationships among hospital ethical climate, nurse-physician collaboration, moral sensitivity, and moral distress. Multiple linear regression with a stepwise approach was employed to identify factors associated with moral distress, which served as the dependent variable. Independent variables included those with *p* < 0.1 in univariate analyses.

## Results

### Characteristics and univariate analysis of participants

A total of 1,292 nurses participated in the study, yielding a response rate of 76.8% (1,292/1,682). Among them, 98.92% were female, with the majority aged 30–39 years. Over two-thirds held a bachelor's degree. In terms of department, 45.59% worked in general pediatric wards, 17.72% in the PICU, and 36.69% in the NICU. Univariate analysis revealed that moral distress scores differed significantly (*p* < 0.05) by department, age, gender, marital status, number of children, years of service, job title, and several subjective perceptions, including adequacy of nurse staffing, job satisfaction, and autonomy in decision-making (Table 1).

**Table 1:** Characteristics and univariate analysis of the moral distress (*n* = 1,292).

Characteristics	Participants, *n* (%)	Moral distress scores median (IQR)	Z/H	*P*
Department				
General pediatric ward	589 (45.59)	60.00 (34.00,76.00)	30.828	0.000*
PICU	229 (17.72)	70.00 (43.00,101.00)		
NICU	474 (36.69)	65.00 (37.00,93.00)		
Age (years)				
<30	485 (37.54)	62.00 (32.00,78.00)	13.486	0.004*
30–39	645 (49.92)	66.00 (39.00,89.00)		
40–49	125 (9.68)	57.00 (39.00,80.00)		
≥50	37 (2.86)	88.00 (45.00,151.00)		
Gender				
Male	14 (1.08)	36.00 (24.00,73.00)	2.006	0.045*
Female	1,278 (98.92)	64.00 (37.00,85.00)		
Educational level				
Associate degree or below	243 (18.81)	65.00 (36.00,78.00)	1.132	0.568
Bachelor's degree	1,020 (78.95)	64.00 (36.50,85.00)		
Master's degree and above	29 (2.24)	60.00 (42.00,103.00)		
Marital status				
Unmarried	324 (25.08)	60.00 (32.00,78.00)	19.599	0.000*
Married	926 (71.67)	64.00 (37.00,86.00)		
Divorced	42 (3.25)	85.00 (51.00,103.00)		
Number of children				
0	424 (32.82)	61.50 (34.00,80.00)	16.578	0.001*
1	639 (49.46)	66.00 (38.50,89.50)		
2	225 (17.41)	63.00 (35.00,83.00)		
3 or more	4 (0.31)	/		
Years in practice				
<5	354 (27.40)	62.00 (38.00,78.00)	8.775	0.032*
5–10	307 (23.76)	64.00 (34.50,80.00)		
>10	631 (48.84)	66.00 (39.50,91.00)		
Professional title				
Primary title	799 (61.84)	61.50 (33.00,80.00)	17.620	0.001*
Middle title	404 (31.27)	68.00 (43.50,90.00)		
Vice-senior title and above	89 (6.89)	68.00 (51.00,103.00)		
Monthly income (CNY)				
≤4,000	132 (10.22)	64.00 (31.00,84.00)	1.571	0.666
4,001–7,000	568 (43.96)	65.50 (35.00,81.50)		
7,001–10,000	439 (33.98)	66.00 (41.00,85.00)		
>10,000	153 (11.84)	57.00 (42.00,90.00)		
Average number of night-shifts (monthly)				
0	228 (17.65)	64.00 (40.00,93.00)	5.731	0.125
1–5	290 (22.45)	68.00 (40.00,85.00)		
6–10	614 (47.52)	63.00 (35.00,82.00)		
>10	160 (12.38)	60.50 (32.50,79.00)		
Whether the number of nurses can meet the demand for clinical work				
Yes	701 (54.26)	60.00 (34.00,80.00)	3.467	0.001*
No	591 (45.74)	69.00 (40.00,89.00)		
Satisfaction with current nursing care				
Yes	920 (71.21)	60.50 (34.00,80.00)	4.825	0.000*
No	372 (28.79)	73.00 (43.00,95.00)		
Availability of clinical decision-making autonomy				
Yes	882 (68.27)	60.00 (33.00,80.00)	5.330	0.000*
No	410 (31.73)	73.00 (46.00,90.00)		
Availability of opportunities to participate in hospital management				
Yes	449 (34.75)	62.00 (36.00,85.00)	0.692	0.489
No	843 (65.25)	65.00 (36.00,84.00)		
Whether trained in ethics				
Yes	427 (33.05)	61.00 (34.00,83.00)	1.673	0.094
No	865 (66.95)	66.00 (38.00,85.00)		

Associate degree or below: requires three years of college education following high school graduation. IQR, interquartile range; CNY, Chinese Yuan; Z, Wilcoxon rank-sum test; H, Kruskal–Wallis H test.

*P<0.05.

### Major study variables descriptions analysis

Table 2 shows the descriptive statistics for the study variables. The median moral distress score among pediatric nurses was 64.00 (IQR: 36.00–84.00), with a mean of 69.71 (range: 16–304). Of the participants, 17 (1.32%) reported having left a healthcare setting due to moral distress, and 106 (8.20%) were considering leaving their clinical position for the same reason. The total scores for hospital ethical climate, moral sensitivity, and healthcare cooperation were 102.55 ± 16.12, 36.50 ± 12.33, and 84.01 ± 15.28, respectively.

**Table 2:** Total scores and scores of various dimensions for the study (*n* = 1,292).

Variables	Score range	Mean ± SD/ median (IQR)
Moral distress	0–336	64.00 (36.00–84.00)
Hospital ethical climate	26–130	102.55 ± 16.12
The relationships of peers	4–20	16.99 ± 2.58
The relationships of patients	4–20	16.51 ± 2.58
The relationships of managers	6–30	25.14 ± 4.38
The relationships of hospital	6–30	24.33 ± 4.24
The relationships of physicians	6–30	19.58 ± 3.62
Moral sensitivity	9–54	36.50 ± 12.33
Moral responsibility and strength	6–36	21.28 ± 7.62
Sense of moral burden	4–24	15.22 ± 5.26
Nurse-physician collaboration	27–135	84.01 ± 15.28
Joint participation in the cure/ care decision making process	12–60	39.84 ± 7.74
Sharing of patient information	9–45	32.30 ± 5.83
Cooperativeness	6–30	11.87 ± 2.46

Moral distress scores exhibited a non-normal distribution and are reported as median (interquartile range).

### Correlation analysis between moral distress, hospital ethical climate, moral sensitivity, and nurse-physician collaboration among pediatric nurses

Spearman’s correlation analysis revealed that lower moral distress was significantly associated with a more positive hospital ethical climate, higher moral sensitivity, and better nurse–physician collaboration (Table 3).

**Table 3:** The correlation between moral distress, hospital ethical climate, moral sensitivity, and nurse-physician collaboration.

Variables	1	2	3	4	5	6	7	8	9	10	11	12	13	14
1 moral distress	1													
2 Hospital ethical climate	−0.358**	1												
3 The relationships of peers	−0.324**	0.890**	1											
4 The relationships of patients	−0.353**	0.895**	0.822**	1										
5 The relationships of managers	−0.356**	0.934**	0.858**	0.807**	1									
6 The relationships of hospital	−0.334**	0.940**	0.788**	0.825**	0.850**	1								
7 The relationships of physicians	−0.319**	0.897**	0.738**	0.750**	0.777**	0.840**	1							
8 Moral sensitivity	−0.319**	0.405**	0.362**	0.400**	0.381**	0.376**	0.365**	1						
9 Moral responsibility and strength	−0.368**	0.479**	0.441**	0.472**	0.470**	0.439**	0.420**	0.925**	1					
10 Sense of moral burden	−0.251**	0.263**	0.226**	0.263**	0.239**	0.243**	0.255**	0.910**	0.717**	1				
11 Nurse-physician collaboration	−0.195**	0.562**	0.490**	0.501**	0.481**	0.527**	0.563**	0.179**	0.216**	0.065**	1			
12 Joint participation in the cure/ care decision making process	−0.172**	0.526**	0.460**	0.466**	0.454**	0.496**	0.536**	0.167**	0.196**	0.167**	0.996**	1		
13 Sharing of patient information	−0.109**	0.554**	0.491**	0.513**	0.472**	0.521**	0.538**	0.179**	0.224**	0.156**	0.943**	0.868**	1	
14 Cooperativeness	−0.192**	0.522**	0.445**	0.444**	0.452**	0.480**	0.548**	0.159**	0.197**	0.160**	0.889**	0.864**	0.797**	1

**p < 0.01 (two-tailed).

### Multivariable analysis of factors influencing moral distress in pediatric nurses

Results of the multiple linear regression analysis are presented in Table 4. The model included participant characteristics with *p* < 0.1 in univariate analysis, along with hospital ethical climate, moral sensitivity, and nurse–physician collaboration as independent variables, and total moral distress score as the dependent variable. Six variables were retained in the final model: department, professional title, gender, hospital ethical climate, moral sensitivity, and nurse–physician collaboration. The model explained 40.2% of the variance in moral distress (R^2^ = 0.402, adjusted R^2^ = 0.398). Collinearity diagnostics indicated no multicollinearity, with tolerance values ranging from 0.383 to 0.964 (all > 0.1) and variance inflation factors (VIF) between 1.012 and 3.632 (all < 5).

**Table 4:** Multiple linear regression analysis for moral distress.

Variable	B	*SE*	*β*	t	*P*	95%CI
constant	140.150	20.421		6.863	0.000	(100.501, 180.711)
Department (ref: general pediatric ward)						
PICU	14.175	2.828	0.118	5.012	0.000	(8.702, 19.801)
NICU	5.579	2.261	0.058	2.468	0.014	(1.382, 10.277)
Professional title (ref: primary title)						
Middle title	6.825	3.296	0.069	2.071	0.039	(−2.810, 11.861)
Vice-senior title and above	16.262	4.683	0.090	3.473	0.001	(3.968, 23.753)
gender (ref: male)	24.255	9.662	0.055	2.510	0.012	(4.320, 42.377)
hospital ethical climate	−1.380	0.077	−0.484	−17.984	0.000	(−1.523, −1.221)
moral sensitivity	−0.982	0.089	−0.263	−11.057	0.000	(−1.151, −0.801)
nurse-physician collaboration	0.593	0.074	0.197	8.006	0.000	(−0.440, −0.732)

Department, professional title, gender, hospital ethical climate, moral sensitivity, and nurse-physician collaboration were associated with moral distress. R = 0.634, R^2^ = 0.402, adjusted R^2^ = 0.398, F = 95.793, *P* < 0.001. SE, standard errors of measurement; B, standard regression coefficient; β, unstandardized regression coefficient.

## Discussion

Moral distress arises when nurses are constrained from implementing appropriate nursing actions or when their clinical practice conflicts with personal and professional values. In this study, the median moral distress score among pediatric nurses was 64.00 (IQR 36.00–84.00), with a mean of 69.71. This score falls within the lower quartile of the total possible range, suggesting an overall low level of moral distress. Nevertheless, all participants reported some degree of moral distress (no returned questionnaires scored zero), indicating its pervasiveness in this population. Notably, the moral distress scores observed here were higher than those reported in national surveys of ICU and oncology nurses in China (28, 29). Pediatric nursing often involves distinct ethical challenges, such as the limited autonomy of child patients and insufficient organizational support for pediatric palliative care (30, 31). These factors may expose pediatric nurses to more frequent and complex ethical dilemmas compared to nurses in other specialties, potentially explaining their elevated moral distress. However, the moral distress level in our sample remained lower than that reported among pediatric nurses in Australia, Iran, and the United States (16, 32, 33). Regular ethics education and training can enhance nurses’ ability to recognize ethical conflicts, strengthen their moral agency, and increase confidence in making ethically sound decisions, thereby reducing moral distress (34). In this study, however, 66.95% of participants had not received such training. The moderate level of moral sensitivity observed may further indicate a diminished acuity in identifying ethical conflicts—a necessary precursor to experiencing moral distress. Although the moral distress level among Chinese pediatric nurses is relatively lower than that in some international reports, it remains higher than that of their domestic counterparts in ICU and oncology settings. Thus, targeted organizational and educational interventions are warranted to mitigate moral distress in this vulnerable group.

Moral distress significantly predicts nurses’ intention to leave and may exacerbate turnover among frontline clinical staff (35). In this study, 8.20% of pediatric nurses reported considering leaving their positions due to moral distress, which is higher than the rate of approximately 6% reported in a Nordic pediatric oncology setting by Ventovaara et al. (17). This issue is particularly concerning given the existing national shortage of pediatric nurses in China. Current data indicate there are 1.5 pediatric healthcare professionals per 10,000 population, comprising 0.6 physicians and 0.7 nurses, resulting in a physician-to-nurse ratio of approximately 1:1.2 (36), far below the WHO-recommended standard of 1:3 (37). Against the backdrop of an aging population and the implementation of China's “three-child policy” to address demographic challenges, pediatric care resources are increasingly strained, and access to care for children has become more difficult. In this context, reducing moral distress-related attrition among pediatric nurses is critically important.

Nurses in the PICU and NICU exhibited higher moral distress than those in general pediatric wards, aligning with findings from prior studies (15, 16, 38). The more critical conditions and poorer prognoses of children in intensive care units likely expose nurses to more frequent ethically challenging situations. Compounding this, pediatric ICU nursing workloads are comparable to or exceed those in adult ICUs, yet nurse staffing is often insufficient. As reported by Lian in 2022 (39), the actual nurse-to-patient ratio in PICUs was 1:3.33, markedly lower than the recommended 1:1.72. Rochefort et al. (40) further indicated that each additional patient per nurse increases the probability of care deficits by 70%. Missed nursing care was a predictive factor that negatively affected moral distress (41). Under such staffing constraints, nurses experience increased workloads, leading to postponed or omitted care. The inability to deliver optimal care in the child's best interest heightens moral distress. These findings underscore the need for nursing managers to enhance psychological support for PICU and NICU nurses and optimize shift-based staffing, particularly during night shifts, to alleviate work-related pressure and mitigate moral distress.

Nurses with middle and senior professional titles reported more severe moral distress than those with junior titles. This finding contrasts with Arash's study of Iranian nurses, which found that greater work experience was associated with reduced moral distress (42). This discrepancy may be attributed to differences in cultural context, organizational structures, clinical processes, and research population characteristics. Nurses holding higher titles generally have more years of clinical experience. Through extended practice, they develop more mature professional values and acquire sharper clinical judgment regarding treatment, care, and prognosis in pediatric patients. This enables them to better identify futile care and advocate for the child's best interests. As a result, they may become more sensitive to ethical conflicts arising from misalignment between care and professional values. Furthermore, with increasing years of practice, senior nurses may accumulate moral residue from past morally distressing situations. When faced with new ethical challenges, this residual distress can amplify their response, leading to more intense moral distress (43). Therefore, greater support should be provided to nurses with higher titles and longer service, including ethics training and professional psychological counseling tailored to their experience level. Gender was also identified as a factor influencing moral distress levels, with female pediatric nurses reporting significantly higher scores than their male counterparts. This finding aligns with previous studies by Dryden-Palmer in 2020 (14) and Mathews in 2023 (44). According to the China Health Statistics Yearbook (https://www.nhc.gov.cn/mohwsbwstjxxzx/tjzxtjsj/tjsj_list.shtml), although nursing remains a female-dominated profession, the proportion of male nurses has been gradually increasing, accounting for 3.8% of all registered nurses in China by the end of 2024. In the present study, however, male pediatric nurses constituted only 1.08% of the sample, which may limit the generalizability of the gender-based findings. Future studies should aim to include more male pediatric nurses to better clarify the role of gender in moral distress.

Hospital ethical climate reflects how an organization addresses ethical issues in healthcare and nurses’ approaches to ethical dilemmas, their decision-making, and subsequent actions (45). In this study, the hospital ethical climate emerged as a key factor influencing moral distress: the more positive the perceived ethical climate, the lower the level of moral distress among pediatric nurses. This finding aligns with reports from pediatric oncology and ICU settings (46, 47). A stronger perceived ethical climate helps nurses better identify with the hospital's ethical environment and professional values, enabling them to provide safer, more effective care to children, thereby reducing the likelihood of moral distress. Moreover, in situations involving ethical conflicts, a supportive ethical climate provides nurses with institutional backing and trust from administrators, colleagues, and patients, which may mitigate the frequency and severity of moral distress (48). Therefore, nursing management fosters an organizational culture that supports ethical practice and cultivates a positive ethical climate at the system level.

Moral sensitivity serves as a prerequisite for ethical behavior in nursing, enabling nurses to better perceive patients’ actual needs and respond more appropriately to clinical ethical issues (49). In this study, moral sensitivity was negatively associated with the level of moral distress among pediatric nurses—a result consistent with findings from a study of new nurses by Xu et al. (50). Nurses with higher moral sensitivity were more attuned to ethical issues and conflicts in clinical practice, demonstrated greater confidence in making and implementing ethical decisions, and were better able to apply ethical principles, thereby reducing moral distress. In contrast, nurses with lower moral sensitivity may struggle to recognize ethical conflicts or empathize with vulnerable patients, increasing the risk of acting in ways that are not aligned with patients’ best interests and subsequently experiencing moral distress. Therefore, nursing managers should implement practical strategies to enhance moral sensitivity among pediatric nurses, such as establishing ethics support teams and providing ongoing ethics training (48).

Regression analyses in this study indicated that higher ratings of healthcare cooperation were associated with more severe moral distress, a finding that contrasts with reports by Kim in 2022 (51) and Hou in 2021 (52), which linked effective collaboration to enhanced autonomy, improved care quality, and reduced moral distress. It should be noted that correlation analysis revealed only a weak negative association between moral distress and nurse-physician collaboration (r = −0.195). This suggests that the relationship may be more complex than a simple linear association. One possible explanation is that in environments with closer collaboration, nurses may gain a clearer understanding of patients’ treatment plans and prognosis, which can heighten their perception of providing futile care. This, in turn, may intensify moral distress when nurses must continue life-sustaining treatments they perceive as medically inappropriate (28). From a clinical perspective, fostering structured collaboration while strengthening nurses’ involvement in decision-making could help mitigate moral distress stemming from external constraints. Similarly, improving interprofessional communication may alleviate distress related to internal ethical conflicts. Healthcare managers should thus promote collaborative practices tailored to clinical realities, establish ethics consultation services and psychological support teams, and encourage open team-based reflection to address moral distress from multiple perspectives (53).

### Potential limitations

This study has several limitations. First, the cross-sectional design precludes causal inference. The lack of longitudinal follow-up also prevents an understanding of how moral distress evolves over time among pediatric nurses. Additionally, the study focused primarily on the current status and influencing factors of moral distress, without employing qualitative methods to explore nurses’ lived experiences and coping strategies in depth.

Second, contrary to previous studies, more experienced nurses in this sample reported higher levels of moral distress. This may be explained by the accumulation of “moral residue”—the lingering psychological burden from prior ethical conflicts—which can heighten sensitivity to subsequent distressing events. Moral distress is not a competence matter, but it is linked to the barriers to performing well. Therefore, further validation among senior nurses is warranted, and future research could also examine the effectiveness of interventions (e.g., ethical support, rounds, or other support mechanisms) in reducing moral distress in this population.

Third, the absence of data on birthplace and urban/rural educational background limited examination of how mobility-related cultural factors—such as interprovincial migration to Sichuan—may influence moral distress. The lower moral distress levels observed in this sample compared to Western studies also point to potential cultural influences that require further investigation.

Fourth, this study focused only on pediatric nurses in Sichuan Province, China. Thus, the generalizability of findings to other regions or healthcare professionals is limited. In future studies, we will further investigate moral distress across diverse regions and among different healthcare professionals.

## Conclusion

Moral distress is prevalent among pediatric nurses in Sichuan Province, China, and its impact on workforce stability warrants attention. Factors significantly associated with moral distress included department, professional title, gender, hospital ethical climate, moral sensitivity, and nurse-physician collaboration.

## Data Availability

The original contributions presented in the study are included in the article/Supplementary Material, further inquiries can be directed to the corresponding author.
