# Supplementary material for: Moral distress among pediatric nurses: a cross-sectional study from Sichuan, China
Source: Front Pediatr. 2026 Apr 29;14:1787502. doi: 10.3389/fped.2026.1787502 (PMC13168100; doi:10.3389/fped.2026.1787502)
Supplement: Supplementary file 1 [file Datasheet1.pdf]

护士道德困境影响因素的系统评价*

黎丽[1],宋锦平[2],唐梦琳[1],冯梅[1]
(1 四川大学华西护理学院/四川大学华西医院重症医学科,四川成都,610041;
2 四川大学华西医院护理部,四川成都,610041)

[摘要] 目的 系统描述和分析护士道德困境发生的影响因素。方法 计算机检索英文数据库PubMed,Embase,CINAHL,Web of Science,Medline(Ovid)及中国知网期刊数据库(China National Knowledge Infrastructure,CNKI)、万方数据库(WANGFANG Date)、维普中文科技期刊数据库(China Science and Technology Journal Database,VIP)、中国生物医学文献数据库(Chinese biomedical literature service system,SinoMed),从建库至2021年10月31日发表的有关护士道德困境影响因素横断面研究的相关文献。根据文献纳入和排除标准进行文献筛选、质量评价和资料提取,采用定性分析的方法归纳总结研究结果。结果 纳入21项研究,归纳出4类影响护士道德困境因素,即社会人口学因素及组织环境、职业态度、心理特质因素,其中良好的组织环境和职业态度可降低护士道德困境的发生,不同心理特质的护士道德困境的表现不同,而社会人口学部分因素作用不一致。结论 护士道德困境的影响因素众多,部分因素的作用存在不同的评价,未来仍需开展纵向研究,以进一步探讨其影响因素。

[关键词] 护士;道德困境;影响因素;系统评价
[中图分类号] R47 [文献标识码] A [文章编号] 1671-8283(2022)12-0062-08 [DOI] 10.3969/j.issn.1671-8283.2022.12.010

Influencing factors of nurses´ moral distress: a systematic review

Li Li[1], Song Jinpi[2], Tang Menglin[1], Feng Mei[1] //Modern Clinical Nursing,-2022,21(12):62.
(1.West China School of Nursing, Si chuan University/Department of Critical Care Medicine, West China Hospital, Sichuan University; 2. Department of Nursing Administration, West China Hospital, Sichuan University, Chengdu, 610041, China.)

[**Abstract**] **Objective** To investigate the factors influencing nurse´ moral distress. **Methods** We searched for the literatures via the databases such as PubMed, Embase, CINAHL, Web of Science, Ovid (Medline), CNKI, WANGFANG Date, VIP and CBM published from the inception to October 31, 2021, having the papers on nurses´ moral distress included in the study. The included papers were sorted out with an inclusion and exclusion standard, followed by quality assessment and data extraction independently by two researchers. The results were analyzed and summarized using qualitative analysis. **Results** A total of 21 papers were included in our study. Four influencing factors were extracted, e.g., socio-demographic factors, organizational environment, professional attitudes, and psychological traits. Among them, the factors of organizational environment and professional attitudes could reduce the occurrence of ethical distress among nurses, the factor of psychological traits influenced their ethical distress in different ways, and the socio-demographic factor played a variant role in the stress among the nurses. **Conclusions** Many factors are associated with nurse´ moral distress. The evaluations over the role of some factors in the stress are different. Longitudinal studies are needed to explore the influencing factors of nurses´ moral distress.

[**Key words**] nurses; moral distress; influencing factors; systematic review

随着医学模式转变、医疗技术进步以及多元文化价值观冲突,医护人员在临床实践中将面对愈来愈多且复杂的伦理问题[1],其中由于内部和外部各种原因的限制,使个体无法做出符合自身信念和价值的措施,从而产生一种受挫、无力的消极情感体验,被称之为“道德困境”[2]。美国重症护士协会(American Association of Critical-Care Nurses,AACN)

[基金项目] *本课题为四川省卫生健康委员会科研课题普及项目,项目编号为19PJ241。
[收稿日期] 2022-04-07
[作者简介] 黎丽(1996-),护师,硕士在读,主要从事儿科护理、护理管理工作。
[通信作者] 宋锦平,主任护师,硕士,E-mail:jinpings210@163.com。

(C)1994-2023 China Academic Journal Electronic Publishing House. All rights reserved. http://www.cnki.net

在一项公开发布的政策声明[3]中,将道德困境称为“医疗环境中经常被忽视的问题”,并提出“4A”法则以识别和处理道德困境。在医疗环境中,护士群体较其他医务群体发生道德困境的频率更为普遍、程度更为严重[4],这不仅会影响护士身心健康及临床护理质量,甚至会导致护士产生职业倦怠及离职意愿,进而加重护理人力资源短缺[5]。缓解护士道德困境是护理领域持续关注并亟待解决的问题之一[6]。护士道德困境有效应对的核心在于分析其影响因素,以便制定有针对性的干预措施。本研究通过对文献检索与分析发现,虽然相关学者已开展了不同执业环境下护士道德困境的现况调查及影响因素研究,但缺乏对其全面系统的归纳与总结。因此,本研究旨系统分析影响护士道德困境水平的相关因素,为我国护理管理者建立健康伦理氛围工作环境,提升临床护理质量,降低护士离职率提供理论支持,为后续探究护士道德困境干预措施提供参考依据。

1 资料与方法

1.1 检索策略

计算机检索英文数据库 PubMed、Embase、CINAHL、Web of Science、Medline(Ovid)及中文数据库的万方数据库(WANGFANG Date)、中国知网期刊数据库(China National Knowledge Infrastructure,CNKI)、维普中文科技期刊数据库(China Science and Technology Journal Database,VIP)、中国生物医学文献数据库(Chinese biomedical literature service system,SinoMed),检索时间均从建库至 2021 年 10 月 31 日。采用自由词加主题词结合检索的方法,以“ethics, nursing/ethical dilemma/moral distress/moral dilemma/Ethic Dilemma/ethical dilemma/nurses/nurs*”为英文检索词,以“道德困境/道德困扰/护士/护理人员/临床护士”为中文检索词进行文献检索。中文检索式以中国知网为例:(SU="道德困境"OR SU="道德困扰") AND (SU="护士"OR SU="临床护士"OR SU="护理人员"),英文检索式以 PubMed 为例:((nurses [MeSH Terms]) OR (nurs* [Title/Abstract])) AND ((((moral distress [Title/Abstract])OR (moral dilemma[Title/Abstract])) OR (ethic dilemma [Title/Abstract])) OR (ethical dilemma[Title/Abstract]))。

1.2 纳入与排除标准

纳入标准:①研究主题与道德困境密切相关;②研究对象为护理工作者;③研究类型为横断面调查;④研究工具包含道德困境相关评估量表;⑤文章语种为中文和英文;⑥公开发表在学术期刊上的原始研究。排除标准:①联系作者后数据仍不完整或无法获取全文;②同一调查重复发表;③研究设计未涉及道德困境影响因素分析的文献。

1.3 文献筛选和资料提取

文献筛选过程由 2 名研究者独立完成,包括 4 个阶段:①经 EndNote 去除重复题录后,从剩余文献中随机抽取 15 篇进行预筛选,以确保研究者对文献纳入排除标准的统一理解;②通过阅读文题进行初筛,剔除明显不符合标准的文献;③进一步通过阅读摘要和全文进行复筛,明确文献是否纳入;④将筛选后的文献进行交叉核对,当遇到分歧时,由第三方或小组讨论协商解决。资料提取过程由 2 名研究者独立完成,提取主要内容包括作者、发表年份、国家、研究类型、研究对象、抽样方法、样本量、研究工具、统计学方法、主要研究结果。

1.4 文献质量评价

由 2 名经过循证培训的研究人员独立进行文献质量的评价,如有分歧,通过协商或寻求第 3 方意见解决。采用澳大利亚 JBI 循证卫生保健中心分析性横断面调查研究(analytical cross-sectional study)的评价工具[7]对纳入文献进行质量评价。2 名研究人员对 8 个评价项目做出“是”“否”“不清楚”不适用的判断,并根据文章整体质量定性评价为 A、B、C 3 个级别。完全满足质量评价标准,发生偏倚的可能较小(A 级);部分满足质量评价标准,发生偏倚的可能性为中等(B 级);完全不满足评价标准,发生偏倚可能性较高(C 级)。

1.5 统计学方法

本研究使用 Endnote 软件进行文献管理,Excel 软件对文献资料数据进行整理和分析。由于本研究中同一结局指标影响因素差异较大,故无法对 OR 值进行合并,因此采用定性描述的分析方法对结果进行分类归纳和描述。

(C)1994-2023 China Academic Journal Electronic Publishing House. All rights reserved. http://www.cnki.net

2 结果

2.1 文献检索结果及质量评价结果

本研究初步检索出相关文献 5741 篇,通过软件剔重、文题初筛、摘要及全文复筛后初步纳入文献 25 篇,对文献进行质量评价后,排除 4 篇 C 级文献,最终纳入文献共 21 篇[8-28],其中有 13 篇文献[10-11,13,15-16,19-23,26-28]质量为 A 级,8 篇文献[8-9,12,14,17-18,24-25]质量为 B 级。文献筛选流程及结果见图 1。纳入文献的基本特征及方法学质量评价结果见表 1。

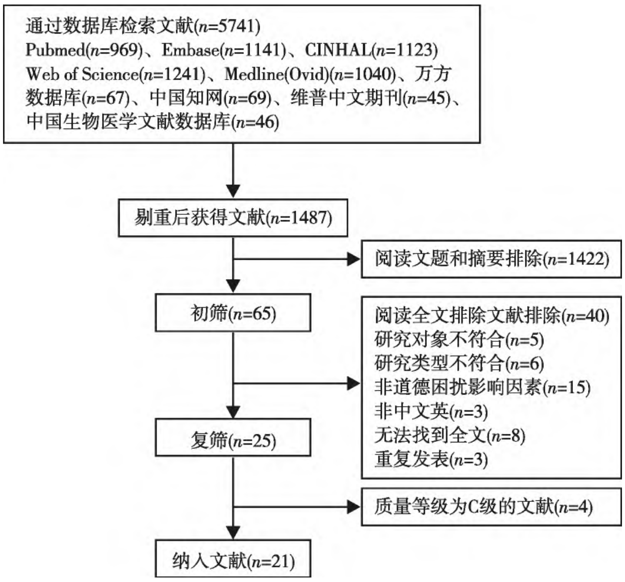

图 1 文献筛选流程图

2.2 纳入文献的基本信息

纳入文献的发表时间为 2011 年-2021 年,其中 57.1%的文献于近 3 年发表;研究对象样本量为 93~2852 例,共计 11614 例;抽样方法中便利抽样使用最多(66.7%);道德困境测评工具以 Corley 等研发的道德困境量表(moral distress scale,MDS)及其修订版本(MDS-R 和 MMD-HP)为主,剩余文献 1 篇使用了道德困境温度计(the moral distress thermometer,MDT),1 篇使用了巴西护士道德困境量表(brazilian scale of moral distress in nurses,EDME-Br),1 篇使用了精神科护士道德困境量表(moral distress scale for psychiatric nurses,MDSP)。具体结果详见表2。

2.3 护士道德困境影响因素

2.3.1 社会人口学因素 纳入文献中共 14 篇文献[8-16,19,22-24,26]涉及社会人口学因素,包括护士年龄、性别、学历、科室、职称、职务、合同性质、工作年限、月收入、婚姻状况、子女情况、每月夜班数,但不同文献呈现的研究结论不一致。①年龄。有 12 篇文献[8-14,16,19,22-24]分析了年龄对护士道德困境水平的影响,其中 8 篇文献[8,11-14,16,23-24]结果表明年龄越大护士道德困境水平越高,但其余 4 篇文献[9-10,19,22]结果表明年龄对护士道德困境无影响。②性别。有 5 篇文献[10-12,22,24]探讨了性别与护士道德困境的相关性,其中 2 篇文献[10-11]结果表明性别对护士道德困境水平无影响,3 篇文献[12,22,24]结果表明女性在医疗环境中

表 1 文献质量评价结果 (n=21)

纳入文献	a	b	c	d	e	f	g	h	质量等级
WU et al[8]	是	是	是	是	是	否	是	是	B
SIRILLA et al[9]	是	是	是	是	不清楚	不清楚	是	是	B
张敏等[10]	是	是	是	是	是	是	是	是	A
沈志莹等[11]	是	是	是	是	是	是	是	是	A
罗昌春等[12]	是	是	否	是	是	是	是	是	B
武宁等[13]	是	是	是	是	是	是	是	是	A
ANNETTE et al[14]	是	是	是	是	不清楚	不清楚	是	是	B
WOLCOTT et al[15]	是	是	是	是	是	是	是	是	A
HOU et al[16]	是	是	是	是	是	是	是	是	A
VENTOVAARA et al[17]	是	是	是	是	不清楚	不清楚	是	是	B
RAMOS et al[18]	是	是	是	是	不清楚	不清楚	是	是	B
PROMPAHAKUL et al[19]	是	是	是	是	是	是	是	是	A

(C)1994-2023 China Academic Journal Electronic Publishing House. All rights reserved. http://www.cnki.net

(续表 1)

纳入文献	a	b	c	d	e	f	g	h	质量等级
OHNISHI et al[20]	是	是	是	是	是	是	是	是	A
YEGANEH et al[21]	是	是	是	是	是	是	是	是	A
PALMER et al[22]	是	是	是	是	是	是	是	是	A
HILER et al[23]	是	是	是	是	是	是	是	是	A
SOLEIMANI et al[24]	是	是	是	是	不清楚	不清楚	是	是	B
WOLF et al[25]	是	是	是	是	不清楚	不清楚	是	是	B
谢敏叶等[26]	是	是	是	是	是	是	是	是	A
SILEN et al[27]	是	是	是	是	是	是	是	是	A
KARANIKOLA et al[28]	是	是	是	是	是	是	是	是	A

注:a.是否清晰界定了研究对象的纳入标准? b.是否详细描述研究对象及研究场所? c.是否采用有效、可信的方法测评暴露因素? d.是否采用客观、标准的方法测评健康问题? e.是否明确了混杂因素? f.是否采取措施控制了混杂因素? g.是否采用有效、可信的方法测评结局指标? h.资料分析方法是否恰当? i.文献质量等级。

表 2 纳入研究基本信息 (n=21)

国家	年份(年)	国家	研究对象	抽样方法	样本量(n)	研究工具	统计学方法	影响因素
WU et al[8]	2018	中国	护士	便利抽样	465	MDS-R	方差分析、相关性分析、多元回归分析	A:①②③④⑤⑥⑦⑧ B:①②③⑬
SIRILLA et al[9]	2017	美国	护士	便利抽样	329	MDS	方差分析、相关性分析、多元回归分析	A:③⑦⑬ B:③
张敏等[10]	2020	中国	ICU 护士	便利抽样	242	MDS-R	相关性分析、多元回归分析	A:⑦⑩⑪⑫⑯ B:⑦⑭
沈志莹等[11]	2017	中国	综合医院护士	便利抽样	378	MDS-R	独立样本 t 检验、方差分析、多元回归分析	A:①②③④⑤⑥⑦ B:①③⑤
罗昌春等[12]	2019	中国	老年医院护士	便利抽样	291	MDS-R	秩和检验、多元线性回归	A:①②④⑤⑥⑦⑨⑫⑱ B:④⑱
武宁等[13]	2019	中国	危急重症	便利抽样	457	MDS-R	独立样本 t 检验、方差分析、Pearson 相关分析、分层回归分析	A:①④⑤⑦⑩ B:①⑦⑩
ANNETTE et al[14]	2013	美国	ICU 护士	便利抽样	277	MDS	相关性分析、多元线性回归	A:① B:⑯
WOLCOTT et al[15]	2018	美国	ICU 护士	便利抽样	235	MDS	相关性分析、多元线性回归	A:②⑭⑯ B:②⑭
HOU et al[16]	2021	中国	急诊护士	便利抽样	237	MDS-R	t 检验、方差分析、相关性分析、逐步多元线性回归	A:①⑧⑬⑭⑮ B:⑧⑭⑮
VENTOVAARA et al[17]	2021	瑞典	儿科肿瘤护士	整群抽样	93	MDS	秩和检验、相关性分析	A:⑭
RAMOS et al[18]	2020	巴西	护士	非概率抽样	1266	EDME-Br	卡方检验、泊松回归	A:㉑ B:㉑
PROMPAHAKUL et al[19]	2021	印度	护士	整群抽样	462	MMD	t 检验、方差分析(ANOVA)、Pearson 相关分析、多层多元回归分析	A:③⑬ B:⑬
OHNISHI et al[20]	2019	日本	精神科护士	整群抽样	1971	MDSP	t 检验,多群结构方程模型	B:㉑
YEGANEH et al[21]	2019	伊朗	ICU 护士	便利抽样	180	MDS	卡方检验、相关性分析	A:⑲
PALMER et al[22]	2020	加拿大	NICU 和 PICU 护士	整群抽样	2852	MDS	秩和检验、多层线性回归	A:⑦⑨⑱ B:⑱
HILER et al[23]	2018	美国	ICU 护士	便利抽样	327	MDS	t 检验、多元回归分析	A:⑬⑮ B:①⑮⑱
SOLEIMANI et al[24]	2019	伊朗	护士	便利抽样	193	MDS	方差分析、相关分析	A:①②⑨⑬
WOLF et al[25]	2019	美国	护士	便利抽样	167	MDT	t 检验、秩和检验、相关性分析	A:⑳
谢敏叶等[26]	2014	中国	护士	整群抽样	387	MDS-R	多元逐步回归分析	A:②④
SILEN et al[27]	2011	瑞典	护士	整群抽样	249	MDS	秩和检验、相关性分析、logistic 回归	B:⑭
KARANIKOLA et al[28]	2014	意大利	ICU 护士	便利抽样	556	MDS	t 检验、方差分析、相关性分析	A:⑬⑰⑱

注:中文版护士道德困境量表(moral distress scale-R,MDS-R);道德困境量表(moral distress scale,MDS);道德困境温度计(The moral distress thermometer,MDT);巴西护士道德困境量表(Brazilian scale of moral distress in nurses,EDME-Br);精神科护士道德困境量表(moral distress scale for psychiatric nurses, MDSP)。A 为单因素分析;B 为多因素分析;①年龄;②学历;③科室;④职称;⑤职务;⑥合同性质;⑦工作年限;⑧月收入;⑨性别;⑩婚姻状况;⑪子女状况;⑫每月夜班数;⑬离职意愿;⑭医院伦理氛围;⑮护理工作环境;⑯心理授权;⑰医护合作;⑱职业主观感受;⑲职业自主性;⑳姑息护理能力感知;㉑道德敏感性。

(C)1994-2023 China Academic Journal Electronic Publishing House. All rights reserved. http://www.cnki.net

经历的道德困境较男性更严重。③学历。有8篇文献[8-12,15,24,26]讨论了学历对护士道德困境的影响,其中2篇文献[9-10]结果表明不同学历护士道德困境水平无差异,5篇文献[8,11-12,15,24]结果表明护士学历越高道德困境水平越高,而有1篇文献[26]结果显示护士教育水平越高,其经历道德困境的程度越轻。④科室。有4篇文献[8-9,11,19]研究结果显示在急诊、ICU工作的护士道德困境水平最高。⑤职称和职务。有6篇文献[8,10-13,26]分析了职称对护士道德困境水平的影响,其中1篇文献[10]结果表明职称与护士道德困境无相关性,4篇文献[8,11-13]结果表明护士职称越高道德困境水平越高,仅1篇文献[26]研究结论恰好相反,即职称越高护士道德困境水平越低。有4篇文献[8,11-13]结果表明,护士职务越高更容易在临床实践中积累道德困境。⑥合同性质。有5篇文献[8,10-12,22]分析了不同合同性质对护士道德困境水平的差异,其中2篇文献[8,22] 结果显示道德困境与合同性质无关,3篇文献[10-12]结果显示正式在编护士较派遣制护士道德困境水平更高。⑦工作年限、月收入。有9篇文献[8-13,15,19,22]分析了护士工作年限对道德困境的影响,其中5篇[8-12]结果表明护士工作年限越大道德困境水平越高,1篇[22]结果表明工作年限大于30年的护士道德困境水平高于工作不满1年的护士,1篇结果[13]表明工作年限为6~9年的护士道德困境水平最高,2篇[15,19] 结果表明护士工作年限对道德困境无影响。有2篇文献[8,16]表明护士月收入越高道德困境水平越高。⑧子女情况、婚姻状况。有1篇文献结果[10]表明护士子女数越多道德困境水平越高。有2篇文献[10,13]分析了不同婚姻状况对护士道德困境的影响,其中1篇结果[10]指出婚姻状况对护士道德困境无影响,1篇[13]表明已婚护士道德困境水平高于未婚护士。⑨每月夜班数。有2篇研究[10,12]分别分析了每月夜班数对护士道德困境的影响,其中1项研究[10]认为,每月夜班数与道德困境水平无相关性,1项研究结果[12]表明每月夜班数为1~3个的护士其道德困境水平较不上夜班的护士和每月夜班数大于3个的护士更高。

2.3.2 组织环境 纳入文献中共9篇文献[8,10,13,15-17,25,27-28]涉及组织环境因素,包括医院伦理氛围、护理工作环境、医护沟通与合作、开展临终关怀培训。①医院伦理氛围作为护士在临床实践中处理工作相关伦理问题的感知,会影响其对伦理问题的态度和处理方式。有5篇文献[10,15-17,27]结果表明医院伦理氛围水平会影响护士经历道德困境,即当护士感知医院伦理氛围的水平越高,其发生道德困境的频率和严重程度会更低。②护理工作环境是指在提供护理服务时,通过授权使护士获得更多自主性、责任感。有3项研究[8,13,16]采用不同的测量工具分析了护理工作环境对护士道德困境的影响,研究结果显示,适度授权护士参与医院事务决策、促进医护一体化团队协作、保证实践环境中人力物力充足、积极开展临终关怀培训、护理管理者具有较强的领导力等健康的护理工作环境可降低护士道德困境的发生。③通过医护沟通与合作可增强医护之间的信任感。仅有1项研究[28]分析了医护合作与道德困境的相关性,结果显示医生与护士通过开放的沟通与协作,可改善医疗服务质量及增强医护信任感,进而降低护士道德困境水平。④开展临终关怀培训。有1篇文献[25]结果表明积极开展姑息护理继续教育,能提升护士姑息治疗的核心能力,但却使护士更容易遭受更大程度的道德压力和道德困境。

2.3.3 职业态度 纳入文献中共7篇文献[9,12,16,21,23-24,28]分析了不同职业态度对护士道德困境水平的影响,包括是否存在离职意愿、目前工作满意度、职业认同感、职业自主性。有5项研究[9,16,23,24,28]结果表明存在离职倾向或曾今有过离职经历的护士,其道德困境水平更为严重。有3项研究[12,23,28]结果显示,当护士对护理工作现状不满意或职业认同感较低时,会更容易经历高水平的道德困境。有1项研究结果[21]表明护士职业自主性与道德困境之间呈显著负相关,可能的原因是当护士职业自主权更高时,会产生更多的道德勇气积极应对道德冲突。

2.3.4 心理特质 纳入文献中共5篇文献[10,14-15,18,20]分析了个体心理特质差异对护士道德困境的影响,包括护士心理授权、道德敏感性。心理授权是指护士感知到工作意义、自我效能感、自主性和工作影响的内在动力。研究结果[10,14-15]显示,当护士心理授权程度减弱时,其道德困境水平随之升高。原因是心理授权水平越高,护士的自我效能感、自主性越强,克服道德困境的能力也随之增强。道德敏感性是护士

(C)1994-2023 China Academic Journal Electronic Publishing House. All rights reserved. http://www.cnki.net

识别道德冲突的能力，是对何为道德问题的清晰认识。有两项研究[18,20]分析了道德敏感性对护士道德困境水平的影响，研究结果显示，道德敏感性水平高的护士能识别和感知道德问题，但却缺乏应对道德冲突的能力，因此会经历更严重的道德困境。

3 讨论

3.1 护士道德困境水平受多种因素的影响

自 1984 年 JAMETON[29]在《护理实践：伦理问题》一书中首次提出道德困境现象以来，该伦理问题一直是护理学、医学与哲学领域的研究热点，而我国于 2011 年才陆续开展相关研究[30]，无论是研究广度和深度均有待提升。虽部分研究[31-32]已证实，道德困境会影响我国护士的身心健康，甚至造成护士离职率升高，但对护士道德困境影响因素的全面分析和干预方案的循证构建相对缺乏。本研究结果显示，影响护士道德困境水平的相关因素复杂多样，主要涉及社会人口学、组织环境、职业态度、心理特质 4 个方面。

3.1.1 影响护士道德困境水平社会人口学因素众多，结论尚不一致 一般来说年龄越大[8,11-14,16,23-24]、工作年限越长[8-12]、学历[8,11-12,15,24]和职称[8,11-13]越高的护士，其道德困境水平越高，但也有学者认为年龄[9-10,19,22]、工作年限[15,19]、学历[9-10]和职称[10]对护士道德困境无影响，也有的学者持相反的意见，认为护士学历[26]和职称[26]越高，其经历道德困境的程度越轻。性别对护士道德困境水平影响也存在分歧，有学者[10-11]认为，性别对护士道德困境水平并无影响，但有的学者[12,22,24]却认为，女性在医疗环境中经历的道德困境较男性更严重。职务、经济收入及子女数也是影响护士道德困境水平的因素，护士职务越高更容易在临床实践中积累道德困境[8,11-13]，护士月收入越高[8,16]和子女数越多[10]的护士，其道德困境水平越高。另外，护士夜班数也会影响其道德困境水平，每月夜班数为 1~3 个的护士其道德困境水平最高[12]。由于急诊、ICU 护理工作的特殊性，因此在急诊、ICU 工作的护士道德困境水平最高[8-9,11,19]。

3.1.2 良好的组织环境可降低护士道德困境的发生 良好的组织环境可使护士在工作中获得成就感和满足感，从而以更加积极的态度对患者实施护理。本研究纳入文献中涉及组织环境因素包括医院伦理氛围、护理工作环境、医护沟通与合作、开展临终关怀培训。相关学者认为，护士感知医院伦理氛围越高，其发生道德严重程度会更低[10,15-17,27]；健康的护理工作环境可降低护士道德困境的发生[8,13,16]；加强医护沟通与合作[28]和开展姑息护理继续教育[25]可降低护士道德困境水平。因此，护理管理者应构建积极的医院伦理氛围、促进良好的医护沟通协作、营造健康的护理实践环境有助于减轻护士道德困境的发生。

3.1.3 不良职业态度的护士道德困境水平较高 护士职业态度是人们对护士职业的社会地位和作用、护士素质要求和现状、护理劳动特点、护理学科性质和发展等方面的认知、情感和行为倾向[33]。本研究纳入文献中涉及职业态度因素包括离职意愿、工作满意度、职业认同感、职业自主性。相关学者认为，存在离职倾向[9,16,23-24,28]、护理工作现状不满意或职业认同感较低[12,23,28]的护士，其道德困境水平较高，有可能导致护士离职的发生。因此，护理管理者应适度授权护士参与医院管理决策、提升职业自主性、改善工作满意度，从而降低护士因道德困境造成的离职情况发生。

3.1.4 不同心理特质的护士道德困境的表现不同 心理特质是指护士心理活动进行时经常表现出的稳定特点。本研究纳入文献中涉及心理特质因素包括护士心理授权、道德敏感性。相关研究结果显示，心理授权水平高的护士道德困境水平较低[10,14-15]，道德敏感性水平高的护士会经历较严重的道德困境[18,20]。因此，护理管理者应提升护士自身道德应激问题的承受能力和心理授权水平，可缓解道德困境引起的不舒适心理体验。

3.2 研究的局限和未来研究方向

本研究严格遵循 JBI 描述性研究系统评价方法，对护士道德困境的影响因素展开了全面的整合分析，但也存在一定不足：①为保证纳入文献方法学的严谨性，仅检索了公开发表的一次文献，尚未收集“灰色文献”；②只对中英文文献进行检索，缺少对非中英文文献的纳入；③纳入文献均为质量等级处于中等水平的描述性研究，在研究设计、抽样方法、统计分析和质量控制上均存在一定方法学上的局限性；④纳入文献虽使用了经广泛验证的道德困境测

(C)1994-2023 China Academic Journal Electronic Publishing House. All rights reserved. http://www.cnki.net

量工具,但各国学者对工具进行了文化调试和修订,使得各维度间的对比研究较为困难。

本研究结果表明,影响护士道德困境水平社会人口学因素众多,结论尚不一致。分析原因如下:①不同国家的文化背景存在差异,基于不同文化背景的个体对道德困境的理解也存在地域差别;②本研究仅纳入与道德困境相关的横断面研究,研究结论无法分析影响因素与道德困境水平的因果关系,且大部分调查采用便利抽样的方法,可能导致样本缺乏代表性;③不同文献对年龄、工作年限、每月夜班数等连续性变量的分段标准不一致,且部分研究仅采用独立样本 t 检验、方差分析、Pearson 相关分析等单因素分析作为人口学特征的比较,随着研究变量的增加,可能会导致研究结果发生Ⅰ类错误的风险;与此同时,部分影响因素需改进评估工具,如道德敏感性的测量采用了自主设计的问卷分析,医护合作水平测量采用的"协作与护理决策满意度量表"仅适用于重症监护室护士。未来需通过更为普适、客观的测评工具验证道德敏感性、医护合作与道德困境的相关性;采用多中心合作对社会人口学因素中年龄、性别、学历、职称、合同性质、工作年限、婚姻状况、每月夜班数对护士道德困境水平是否有影响及影响程度进一步研究验证。

4 结论

本研究纳入的21篇文献能较全面地反映影响护士道德困境的因素,总结出21个护士道德困境的影响因素,整合为社会人口学因素、组织环境、职业态度、心理特质4个方面,为护理管理者针对性护理措施的开展提供依据。但影响护士道德困境水平的社会人口学因素众多,结论尚不一致,对年龄、工作年限、每月夜班数等连续性变量的分段标准不一致,可能造成研究结果的偏倚;本研究仅纳入横断面研究,且大部分调查采用便利抽样的方法,可能导致样本缺乏代表性;另外,护士道德困境是一个动态变化的过程,未来需长期进行追踪,以进一步探讨护士道德困境的影响因素。

参考文献:

[1] RAINER J,SCHNEIDER J K,LORENZ R A. Ethical dilemmas in nursing:an integrative review[J]. J Clin Nurs,2018,27(19):3441-3446.

[2] MCCARTHY J,GASTMANS C. Moral distress:a review of the argument-based nursing ethics literature[J]. Nurs Ethics,2015,22(1):131-135.

[3] LAMIANI G,BORGHI L,ARGRNTERO,et al. When healthcare professionals cannot do the right thing:a systematic review of moral distress and its correlates[J]. J Health Psychol,2017,22(1):51-67.

[4] HALLY S M,SETTLE M,NELSON B D. Relationship between moral distress and intent to leave a position among neonatal intensive care nurses[J]. Adv Neonatal Care,2021,27(1):1-8.

[5] MCCUE C. Using the AACN framework to alleviate moral distress[J]. Online J Issues Nurs,2010,16(1):9-16.

[6] MORLEY G,JONES C B,LVES J. What is ´moral distress´ in nursing a feminist empirical bioethics study[J]. Nurs Ethics,2020,27(5):1297-1314.

[7] 胡雁,郝玉芳.循证护理学[M].北京:人民卫生出版社,2018:157-174.

[8] WU X Y,ZHAN Y F,CI LF,etal. Moral distress and its influencing factors:a cross-sectional study in China[J]. Nurs Ethics,2018,25(4):470-480.

[9] SIRILLA J,THOMPSON K,YAMOKOSKI T,et al. Moral distress in nurses providing direct patient care at an academic medical center[J]. Worldviews Evid Based Nurs,2017,14(2):128-135.

[10] 张敏,冷雅楠,关志,等.重症监护病房护士道德困境水平现状及其与医院伦理氛围和心理授权的相关性[J].解放军护理杂志,2020,37(2):23-27.

[11] 沈志莹,钟竹青,丁四清,等.长沙市三级甲等综合医院护士道德困境现状及其影响因素分析[J].中国护理管理,2017,17(1):50-54.

[12] 罗昌春,纪冬梅,李海芳,等.北京市某三级老年医院护士道德困境现状及其影响因素分析 [J]. 中国护理管理,2019,19(4):553-558.

[13] 武宁,谭雅琼,李乐之.急危重症护士工作环境对道德困境影响的研究[J].中国护理管理,2019,19(1):87-91.

[14] ANNETTE M.Moral distress and psychological empowerment in critical care nurses caring for adults at end of life[J].Am J Crit Care,2013,22(2):143-151.

[15] WOLCOTT A K,JILL H E,JANINE K,et al.Relationships among palliative care,ethical climate,empowerment,and moral distress in intensive care unit nurses[J].Am J Crit Care,2018,27(4):295-302.

(C)1994-2023 China Academic Journal Electronic Publishing House. All rights reserved. http://www.cnki.net

[16] HOU Y C, TIMMINS F, ZHOU Q, et al. A cross-sectional exploration of emergency department nurses´ moral distress, ethical climate and nursing practice environment[J]. Int Emerg Nurs, 2021, 55: 100972

[17] VENTOVAARA P, SANDEBERG M A, RASANEN J, et al. Ethical climate and moral distress in paediatric oncology nursing[J]. Nurs Ethics, 2021, 28(6): 1061-1072.

[18] RAMOS F R S, BREHMER L C F, DALMOLINGL, et al. Association between moral distress and supporting elements of moral deliberation in nurses[J]. Rev Lat Am Enfermagem, 2020, 28: 1-8.

[19] PROMPAHAKUL C, MALPASS J K, LEBARON V, et al. Moral distress among nurses: a mixed-methods study[J]. Nurs Ethics, 2021, 28(7-8): 1165-1182.

[20] OHNISHI K, KITAOKA K, NAKAHAR J, et al. Impact of moral sensitivity on moral distress among psychiatric nurses[J]. Nurs Ethics, 2019, 26(5): 1473-1483.

[21] YEGANEH M R, POURALLIZADEH M, GHANBARI A, et al. The relationship between professional autonomy and moral distress in ICU nurses of guilan university of medical sciences in 2017[J]. Nurs Pract Today, 2019, 6(3): 133-141.

[22] PALMER K D, MOORE G, MCNEIL C, et al. Moral distress of clinicians in canadian pediatric and neonatal ICUs[J]. Pediatr Crit Care Med, 2020, 21(4): 314-323.

[23] HILER C A, HICKMAN R L, REIMER A P, et al. Predictors of moral distress in a US sample of critical care nurses[J]. Am J Crit Care, 2018, 27(1): 59-66.

[24] SOLEIMANI M A, SHARIF S P, YAGHOOBZADEH A, etal. Spiritual well-being and moral distress among Iranian nurses[J]. Nurs Ethics, 2019, 26(4): 1101-1113.

[25] WOLF AT, WHITE K R, EPSTEIN E G, et al. Palliative care and moral distress: an institutional survey of critical care nurses[J]. Crit Care Nurse, 2019, 39(5): 38-49.

[26] 谢敏叶,任晓碧,赵玲玲,等.护士社会支持对道德困境的影响研究[J].中国护理管理杂志,2014,14(11):773-775.

[27] SILEN M, SVANTESSON M, KJELLSTROMS, et al. Moral distress and ethical climate in a swedish nursing context: perceptions and instrument usability[J]. Journal of Clinical Nursing, 2011, 20(23-24): 3483-3493.

[28] KARANIKOLAM N K, ALBARRAN J W, DRIGO E, et al. Moral distress, autonomy and nurse-physician collaboration among intensive care unit nurses in Italy[J]. J Nurs Manag, 2014, 22(4): 472-484.

[29] JAMETON A. Nursing practice: the ethical issues[M]. Englewood Cliffs, NJ: Prentice Hall, 1984: 1-6.

[30] 孙霞.中文版护士道德困境量表的修订及在护理人员中的应用[D].济南:山东大学,2011.

[31] 姚秀钰,卢鹃鹃,杨一瑶,等.北京三级甲等综合医院急诊科护士道德困境与职业倦怠相关性[J].中国护理管理,2018,18(11):1511-1515.

[32] 赵静静.石河子市三甲医院护士道德困境与工作疏离感相关性研究[D].新疆:石河子大学,2019.

[33] 吴雅文,姜安丽,李树珍.护理学情感教学评价工具的研究[J].中国高等医学教育,2000,13(1):58-60.

[本文编辑:郑志惠]

·编读往来·

禁止一稿多投

“一稿多投”是指作者把自己的一部作品同时或者先后发给不同的出版社或其他媒体,即多次使用同一作品的行为。对科技期刊来说,一篇投稿的录用需要经过初审、外审、定稿等多个流程,同时需要编辑花费大量的时间和精力进行修改,如果作者同时向多个刊物投稿,必将导致大量的重复性劳动和编辑资源浪费,将严重伤害科技期刊和广大作者的利益。敬请各位作者慎重选择投稿刊物,并确定前一次投稿已被退稿后再行改投。

[本刊编辑部]

(C)1994-2023 China Academic Journal Electronic Publishing House. All rights reserved. http://www.cnki.net
