# Supplementary material for: Moral distress among pediatric nurses: a cross-sectional study from Sichuan, China
Source: Front Pediatr. 2026 Apr 29;14:1787502. doi: 10.3389/fped.2026.1787502 (PMC13168100; doi:10.3389/fped.2026.1787502)
Supplement: Supplementary file 2 [file Table1.docx]

**Table 1 Characteristics and Univariate Analysis of the Moral Distress (n = 1292)**

| Characteristics | Participants, n (%) | Moral Distress Scores  Median (IQR) | Z/H | P |
| --- | --- | --- | --- | --- |
| Department |  |  |  |  |
| General pediatric ward | 589（45.59） | 60.00（34.00,76.00） | 30.828 | 0.000^*^ |
| PICU | 229（17.72） | 70.00（43.00,101.00） |  |  |
| NICU | 474（36.69） | 65.00（37.00,93.00） |  |  |
| Age（years） |  |  |  |  |
| ＜30 | 485（37.54） | 62.00（32.00,78.00） | 13.486 | 0.004^*^ |
| 30-39 | 645（49.92） | 66.00（39.00,89.00） |  |  |
| 40-49 | 125（9.68） | 57.00（39.00,80.00） |  |  |
| ≥50 | 37（2.86） | 88.00（45.00,151.00） |  |  |
| Gender |  |  |  |  |
| Male | 14（1.08） | 36.00（24.00,73.00） | 2.006 | 0.045^*^ |
| Female | 1278（98.92） | 64.00（37.00,85.00） |  |  |
| Educational level |  |  |  |  |
| Associate degree or below | 243（18.81） | 65.00（36.00,78.00） | 1.132 | 0.568 |
| Bachelor’s degree | 1020（78.95） | 64.00（36.50,85.00） |  |  |
| Master's degree and above | 29（2.24） | 60.00（42.00,103.00） |  |  |
| Marital status |  |  |  |  |
| Unmarried | 324（25.08） | 60.00（32.00,78.00） | 19.599 | 0.000^*^ |
| Married | 926（71.67） | 64.00（37.00,86.00） |  |  |
| Divorced | 42（3.25） | 85.00（51.00,103.00） |  |  |
| Number of children |  |  |  |  |
| 0 | 424（32.82） | 61.50（34.00,80.00） | 16.578 | 0.001^*^ |
| 1 | 639（49.46） | 66.00（38.50,89.50） |  |  |
| 2 | 225（17.41） | 63.00（35.00,83.00） |  |  |
| 3 or more | 4（0.31） | / |  |  |
| Years in practice |  |  |  |  |
| ＜5 | 354（27.40） | 62.00（38.00,78.00） | 8.775 | 0.032^*^ |
| 5-10 | 307（23.76） | 64.00（34.50,80.00） |  |  |
| ＞10 | 631（48.84） | 66.00（39.50,91.00） |  |  |
| Professional title |  |  |  |  |
| Primary title | 799（61.84） | 61.50（33.00,80.00） | 17.620 | 0.001^*^ |
| Middle title | 404（31.27） | 68.00（43.50,90.00） |  |  |
| Vice-senior title and above | 89（6.89） | 68.00（51.00,103.00） |  |  |
| Monthly income (CNY) |  |  |  |  |
| ≤4000 | 132（10.22） | 64.00（31.00,84.00） | 1.571 | 0.666 |
| 4001-7000 | 568（43.96） | 65.50（35.00,81.50） |  |  |
| 7001-10000 | 439（33.98） | 66.00（41.00,85.00） |  |  |
| ＞10000 | 153（11.84） | 57.00（42.00,90.00） |  |  |
| Average number of night-shifts (monthly) |  |  |  |  |
| 0 | 228（17.65） | 64.00（40.00,93.00） | 5.731 | 0.125 |
| 1-5 | 290（22.45） | 68.00（40.00,85.00） |  |  |
| 6-10 | 614（47.52） | 63.00（35.00,82.00） |  |  |
| ＞10 | 160（12.38） | 60.50（32.50,79.00） |  |  |
| Whether the number of nurses can meet the demand for clinical work |  |  |  |  |
| Yes | 701（54.26） | 60.00（34.00,80.00） | 3.467 | 0.001^*^ |
| No | 591（45.74） | 69.00（40.00,89.00） |  |  |
| Satisfaction with current nursing care |  |  |  |  |
| Yes | 920（71.21） | 60.50（34.00,80.00） | 4.825 | 0.000^*^ |
| No | 372（28.79） | 73.00（43.00,95.00） |  |  |
| Availability of clinical decision-making autonomy |  |  |  |  |
| Yes | 882（68.27） | 60.00（33.00,80.00） | 5.330 | 0.000^*^ |
| No | 410（31.73） | 73.00（46.00,90.00） |  |  |
| Availability of opportunities to participate in hospital management |  |  |  |  |
| Yes | 449（34.75） | 62.00（36.00,85.00） | 0.692 | 0.489 |
| No | 843（65.25） | 65.00（36.00,84.00） |  |  |
| Whether trained in ethics |  |  |  |  |
| Yes | 427（33.05） | 61.00（34.00,83.00） | 1.673 | 0.094 |
| No | 865（66.95） | 66.00（38.00,85.00） |  |  |

*Note. Associate degree or below: requires three years of college education following high school graduation. IQR: interquartile range. CNY: Chinese Yuan. Z: Wilcoxon rank-sum test. H: Kruskal-Wallis H test.*

**Table 2 Total scores and scores of various dimensions for the study (n = 1292)**

| **Variables** | **Score Range** | **Mean±SD/ median (IQR)** |
| --- | --- | --- |
| Moral distress | 0-336 | 64.00 (36.00-84.00) |
| Hospital ethical climate | 26-130 | 102.55 ±16.12 |
| The relationships of peers | 4-20 | 16.99±2.58 |
| The relationships of patients | 4-20 | 16.51±2.58 |
| The relationships of managers | 6-30 | 25.14±4.38 |
| The relationships of hospital | 6-30 | 24.33±4.24 |
| The relationships of physicians | 6-30 | 19.58±3.62 |
| Moral sensitivity | 9-54 | 36.50 ± 12.33 |
| Moral responsibility and strength | 6-36 | 21.28±7.62 |
| Sense of moral burden | 4-24 | 15.22±5.26 |
| Nurse-physician collaboration | 27-135 | 84.01± 15.28 |
| Joint participation in the cure/ care decision making process | 12-60 | 39.84±7.74 |
| Sharing of patient information | 9-45 | 32.30±5.83 |
| Cooperativeness | 6-30 | 11.87±2.46 |

*Note.* *Moral distress scores exhibited a non-normal distribution and are reported as median (interquartile range).*

**Table 3 The correlation between moral distress, hospital ethical climate, moral sensitivity, and nurse-physician collaboration**

|  | 1 | 2 | 3 | 4 | 5 | 6 | 7 | 8 | 9 | 10 | 11 | 12 | 13 | 14 |
| --- | --- | --- | --- | --- | --- | --- | --- | --- | --- | --- | --- | --- | --- | --- |
| 1 moral distress | 1 |  |  |  |  |  |  |  |  |  |  |  |  |  |
| 2 Hospital ethical climate | -0.358^**^ | 1 |  |  |  |  |  |  |  |  |  |  |  |  |
| 3 The relationships of peers | -0.324^**^ | 0.890^**^ | 1 |  |  |  |  |  |  |  |  |  |  |  |
| 4 The relationships of patients | -0.353^**^ | 0.895^**^ | 0.822^**^ | 1 |  |  |  |  |  |  |  |  |  |  |
| 5 The relationships of managers | -0.356^**^ | 0.934^**^ | 0.858^**^ | 0.807^**^ | 1 |  |  |  |  |  |  |  |  |  |
| 6 The relationships of hospital | -0.334^**^ | 0.940^**^ | 0.788^**^ | 0.825^**^ | 0.850** | 1 |  |  |  |  |  |  |  |  |
| 7 The relationships of physicians | -0.319^**^ | 0.897^**^ | 0.738^**^ | 0.750^**^ | 0.777^**^ | 0.840^**^ | 1 |  |  |  |  |  |  |  |
| 8 Moral sensitivity | -0.319^**^ | 0.405^**^ | 0.362^**^ | 0.400^**^ | 0.381^**^ | 0.376^**^ | 0.365^**^ | 1 |  |  |  |  |  |  |
| 9 Moral responsibility and strength | -0.368^**^ | 0.479^**^ | 0.441^**^ | 0.472^**^ | 0.470^**^ | 0.439^**^ | 0.420^**^ | 0.925^**^ | 1 |  |  |  |  |  |
| 10 Sense of moral burden | -0.251^**^ | 0.263^**^ | 0.226^**^ | 0.263^**^ | 0.239^**^ | 0.243^**^ | 0.255^**^ | 0.910^**^ | 0.717^**^ | 1 |  |  |  |  |
| 11 Nurse-physician collaboration | -0.195^**^ | 0.562^**^ | 0.490^**^ | 0.501^**^ | 0.481^**^ | 0.527^**^ | 0.563^**^ | 0.179^**^ | 0.216^**^ | 0.065^**^ | 1 |  |  |  |
| 12 Joint participation in the cure/ care decision making process | -0.172^**^ | 0.526^**^ | 0.460^**^ | 0.466^**^ | 0.454^**^ | 0.496^**^ | 0.536^**^ | 0.167^**^ | 0.196^**^ | 0.167^**^ | 0.996^**^ | 1 |  |  |
| 13 Sharing of patient information | -0.109^**^ | 0.554^**^ | 0.491^**^ | 0.513^**^ | 0.472^**^ | 0.521^**^ | 0.538^**^ | 0.179^**^ | 0.224^**^ | 0.156^**^ | 0.943^**^ | 0.868^**^ | 1 |  |
| 14 Cooperativeness | -0.192^**^ | 0.522^**^ | 0.445^**^ | 0.444^**^ | 0.452^**^ | 0.480^**^ | 0.548^**^ | 0.159^**^ | 0.197^**^ | 0.160^**^ | 0.889^**^ | 0.864^**^ | 0.797^**^ | 1 |

*Note. ** p < 0.01 (two-tailed).*

**Table 4 Multiple linear regression analysis for moral distress**

| **Variable** | **B** | ***SE*** | ***β*** | **t** | ***P*** | ***95%CI*** |
| --- | --- | --- | --- | --- | --- | --- |
| constant | 140.150 | 20.421 |  | 6.863 | 0.000 | (100.501, 180.711) |
| Department (ref: general pediatric ward) |  |  |  |  |  |  |
| PICU | 14.175 | 2.828 | 0.118 | 5.012 | 0.000 | (8.702, 19.801) |
| NICU | 5.579 | 2.261 | 0.058 | 2.468 | 0.014 | (1.382, 10.277) |
| Professional title (ref: primary title) |  |  |  |  |  |  |
| Middle title | 6.825 | 3.296 | 0.069 | 2.071 | 0.039 | (-2.810, 11.861) |
| Vice-senior title and above | 16.262 | 4.683 | 0.090 | 3.473 | 0.001 | (3.968, 23.753) |
| gender (ref: male) | 24.255 | 9.662 | 0.055 | 2.510 | 0.012 | （4.320, 42.377） |
| hospital ethical climate | -1.380 | 0.077 | -0.484 | -17.984 | 0.000 | （-1.523, -1.221） |
| moral sensitivity | -0.982 | 0.089 | -0.263 | -11.057 | 0.000 | （-1.151, -0.801） |
| nurse-physician collaboration | 0.593 | 0.074 | 0.197 | 8.006 | 0.000 | （-0.440, -0.732） |

*Note. Department, professional title, gender, hospital ethical climate, moral sensitivity, and nurse-physician collaboration were associated with moral distress. R=0.634, R²=0.402, adjusted R²=0.398, F=95.793, P<0.001. SE = standard errors of measurement, B = standard regression coefficient, and β = unstandardized regression coefficient.*
